# Supplementary material for: Cisplatin Mouse Models: Treatment, Toxicity and Translatability
Source: Biomedicines. 2021 Oct 7;9(10):1406. doi: 10.3390/biomedicines9101406 (PMC8533586; doi:10.3390/biomedicines9101406)
Supplement: Supplementary file 1 [file biomedicines-09-01406-s001.zip › biomedicines-1384730-supplementary.pdf]

Table S1. Publications reporting cisplatin protocols in tumor-bearing mice published in the period from April 2020 to February 2021. Cisplatin was mostly used as a treatment control of therapeutic and/or toxic effects.

Strain (origin)	Age, sex, N	start	Tumor cells	Cisplatin therapy	Cisplatin protocol	Results	Study Endpoints and humane intervention	ref
Swiss albino (India)	Male, female, N=6	D0	EAC-Ehrlich ascites carcinoma cells 1x10^6 (ip); DLA- Dalton's lymphoma ascites cells (sc: hind limb)	D1	3.5mg/kg ip single dose<br><br>Vs. Annona muricata Linn leaf methanol extracts (antitumor activity evaluation) 10 days (po) daily	Survival due to tumor burden (EAC), cisplatin died D40, control D25; tumor size (DLA) (D13 start, every 3 days up to 42 days) ; cisplatin 86.3% higher inhibition than control;	D40 (EAC)<br>D43 (DLA)<br><br>tumor caused limb dysfunction (1.5 cm^3)	[1]
Nude (USA)	Male, 6-8wk, N=6		Allograft 344SQ and xenograft H460: 0.8x10^6 sc flank<br>non-small cell lung cancer<br><br>experiment repeated 3 times (robustness)	tumor 80-150mm^3 – D0	Cisplatin +etoposide + X-ray irradiation; Low-dose CP<br>Mixture of free CP=1.16mg/kg and ET=1.25mg/kg iv (tail) single dose, followed by fractionated X-ray irradiation (5 Gy x3days) D0 (3h after injection), D1, D2; CPP=2.25mg/kg ; ET:CP/CPP=1:1.8<br>vs etoposide and cisplatin dual drug loaded nanoparticles nanotherapeutics (prodrug approach)	D3:blood:WBC, AST, BUN, Cr (N=2 randomly selected); -all in normal range<br>BW loss due to XrayT<br>Tumor histology: caspase 3, CD-DNA complexes (CP delivery)<br>TS:2cm in diameter- up to D20 after treatment	HEP: BW ↓20%, TS ↑2cm<br>General health deterioration (a vet decision)<br><br>BW, TS: every other day<br>A vet advice when significant? deterioration in general health	[2]
B6(Cg)-Tyr^{c-2J}/J (B6-Albino) (USA)	Male, female, 8-10 wk N=6	D0	(Luc-tdT) Lewis Lung Carcinoma cells 1x10^6 iv (tail) to inoculate cells in the lungs ortothopically (rapid and aggressive tumor growth)<br><br>experiment repeated 3 times (robustness)<br><br>periorbital injection<br><br>randomization into groups before treatment	D7	MTD determined in pilot study on C57BL/6J, periorbital injection (iv) 100µl/20g of cisplain under 2.5% isofluran (n=3) different concentration 3-5 mg/kg;<br>3 cycles of 3x cisplatin every other day+2day recovery between cycles; dose-depended toxicity profile:<br>5 mg/kg: no recovery, BW drop to 80% at day10 (cumulative 15 mg/kg)<br>4mg/kg : drop of BW to 80% at day15 (cumulative 24 mg/kg)<br>3mg/kg: BW to 90% at d10, >80% at d19 (cumulative 27mg/kg)<br>based on BW drop 3mg/kg was chosen;<br>Treatment: 2 cycles of 3x3mg/kg+2 day recovery (cumulative 18mg/kg)<br><br>PLX into lungs under anesthesia 25µL/20g vs therapeutic potential of PLX 3397, a molecule CSF-1R inhibitor alone or combined with cisplatin	Survival (preliminary study) (N=5)<br>D20-D26<br>bioluminescence imaging (BLI) at D0,7,12,19<br>Lungs weigh, tumor nodules from lungs collected and further processed;<br>Lung: M1 (F4/80, CD80), M2 (F4/80, CD206)<br><br>B6 albino mice chosen due to reduced BLI signal through skin and fur in B6 mice	D12<br>D19<br><br>HEP: BW ↓20%, TS,<br>inability to move freely obvious signs of illness, or significant quivering, inability to eat/drink properly<br><br>the behavior observed daily, and clinical signs scored using scores S.aureus [3], BW recorded at least every other day	[4]
nude mice (China)			xenograft oral carcinoma cells ACC83		10mg/kg ip every other day till day 28 (cumulative 140mg/kg) ??<br>Vs role of TGFβ in kidney toxicity and protective effects of ginsenoside Rb3	BW (no drop observed in cis)<br>Kidney: histology HE, TGFb (IHC, WB); mechanism was examined on cell lines	D28	[5]
BALB/c nude mice (China)	Female 4-5 wks, N=8		xenograft lung cancer cells 4549/DDP 7x10^6 sc flank	100mm^3 d0<br><br>randomly divided	2x5mg/kg /week for 3 wks; iv (cumulative 30 mg/kg)<br>Half-life $T_{1/2}$ =11.31h<br>Vs GSH-responsive and pH-responsive cisplatin prodrug - nanotechnology	BW (not shown), ≈ALT, ↑Cr, ≈↑WBC; TS<br>Drug distribution in tissues: kidney> heart, liver, tumor, > spleen, lung	D21	[6]
C57BL/6J,	Female		BALB: Mouse colon		MTD determined by cisplatin nephrotoxicity:	MTD:	D30, D35?	[7]

BALB/cA, C.B-17/Lcr – scid SCID (Japan)	8-12wk		adenocarcinoma cells (CT26) 1.5x10⁶ sc<br><br>SCID: human lung adenocarcinoma cells (A549) 2.5x10⁶ sc<br><br>MTD - healthy mice		3x 3mg/kg ip (d0,3,6) ip (cumulative 9mg/kg) - no KIM-1 (IHC); ≈BUN<br>3x 6mg/kg (d0/3/6) (cumulative 18mg/kg) - ↑KIM-1, ↑BUN<br>BW drop from d4on BALB/cA<br>Tumor bearing mice 2 protocols<br>2x4mg/kg (D2,5) ip (CT26 BALB) (cumulative 8 mg/kg) or<br>3x 3mg/kg (d2,5,8) (A549 SCID) (cumulative 9 mg/kg) – no nephrotoxicity<br>3x 3mg/kg (d6,9,12) or<br>3x 6 mg/kg (d6,9,12) ip with<br>Cilastatin once daily for 7 days<br>Vs megalin endocytic receptor in kidney cilastatin blocked binding of cisplatin to magalin – reduction of AKI<br>Cisplatin+cilastatin combined therapy	d7, WBC; RBC<br>d14 kidney KIM-1; d9 ??<br>TS, BW<br>Kidney: histology, KIM-1 (IHC)	HEP: Tumor 600mm3<br><br>↓Reporting quality!	
SCID (canada)	Females 6-8wks N=3 ?		3 cancer cell lines sc into left flank; cisplatin-sensitive cervical cancer cells (ME-180) 2x10⁶; intermediately cisplatin-resistant lung cancer (A549) 5x10⁶; highly cisplatin-resistant ovarian cancer 6x10⁶ (NIH:OVCAR-3)	200mm³ (ME-180, A549)<br><br>150mm³ (NIH:OVCAR-3)<br><br>D0	3 ×2 mg/kg (cumulative 6 mg/kg) ME-180,<br>3 ×2.5 mg/kg (cumulative 7.5 mg/kg) A549,<br>4 ×2.5 mg/kg (cumulative 10 mg/kg) NIH:OVCAR-3:<br>Treatment every other day<br>Vs. RDM-B (BV10) - Combination therapy to overcome severe toxic side effects (nephro-, neuro-, oto-toxicity) and drug resistance;	TS ; acute toxicity 2 mice from each group 24 h after the last treatment (10 mg/kg)- blood and serum; hepatotoxicity - ALT; ALP (alkaline phosphatase); TBILS (total bilirubin); nephrotoxicity: electrolytes (Na, K, Cl), BUN, Cr,<br>gut toxicity: TUNEL<br>all in normal range except mucosa of the intestine	D30 (ME-180)<br>D40 (NIH:OVCAR-3)<br>D80 (A549)<br><br>HEP: BW ↓20%, TS the longest axis > 17 mm, tumor ulceration, body condition score (BCS) < 2	[8]
BALB/c (France)	N=5	D06 mm³	CT-26 1x10⁶ sc	D10	therapy dose: 5 x 1.5 mg/kg ip (every 3 days) (cumulative 7.5 mg/kg);<br>toxicity dose: single 20mg/kg ip:3 days after kidney, liver, body weight:<br>10mg/kg (fig 6), 20mg/kg, (materials) 30mg/kg (results)!?<br>LDL as nanocarriers of cisplatin to cancer cells or macrophages (HDL)	Therapy; TS every 3 days<br><br>Toxicity : TS↓, BW↓19%<br>Kidney HE↑lesion, ↑caspase-3 (IF)	D25 (therapy)<br><br>D13 (toxicity)<br><br>↓Reporting quality!	[9]
Swiss albino (Egypt)	N=10	D0	Ehrlich Ascites Carcinoma cells (EAC) 2.5 x10⁶ intradermally? on two sides	D7	2mg/kg ip every 3 days for 2 weeks (cumulative 10 mg/kg)	Blood: ↑BUN, ↑Cr, ≈↑*ALT; ≈↑*AST,<br>tumor weight, miRNA122, VEGFR2, CD34 – angiogenesis<br>≈↑* sig but in normal range?	D21	[10]
BALB/c nude (Shanghai	Female 4-5wks N=6		Human ovarian cancer cells (SKOV3)<br><br>cisplatin resistant human ovarian cancer cells (SKOV3- DDP) 2 x10⁶ sc back	50mm³ treatment D0<br><br>tumor 200mm³ imaging	2mg/kg 2x per week for 35 days (5wks) iv (cumulative 20mg/kg)<br><br>multifunctional tumor target nanoparticles for monitoring therapeutic effects by dual-mode imaging and overcoming cisplatin resistance, ultrasound facilitates drug and gene delivery across biological barriers	BW↓, TS every 2 days<br>blood: BUN, Cr, ALT; AST, ALT, CA125<br>systemic toxicity of free Pt, hemolysis of blood 5%<br>tumor: Ki67, Bcl-2, Bax, CytC, caspase9, caspase3; organs HE: heart, kidney, liver, spleen, lung	D36<br><br>TV ↑2000mm³ (control!)	[11]

Balb/c nude (China)	female		Human ovarian cancer cells cisplatin resistant (A2780CP) 1 x10^7 sc flank	tumors palpated D0 randomly divided	4x 3mg/kg iv (on D1,4,7,10) (total 12mg/kg) FSH peptide loaded with HK2 shRNA to selectively suppress HK2 expression in ovarian cancer - nanoparticles	BW, TS, Tumor: HE, IHC: ATP7b, Bax, CytC, caspase3, TUNEL	D13<br><br>TV 1500mm^3 (control) – 2-2.5g	[12]
BALB/c nude (China)	Female20g N=5		Human breast cancer cells (MDA-MB231) 2x10^6 sc	50mm3 D0 randomly divided	cisplatin dose? iv 6x (D0,3,6,9,12,14)<br><br>cisplatin + auraptene in nanogel as synergistic delivery targeted therapy	BW↓30% , TV (max400mm^3), D10 one mouse died Histology: kidney, heart, liver, spleen, lung (alteration only in liver, kidney, heart-fibrous tissue), Blood hemolysis	D16<br><br>↓Reporting quality!	[13]
BALB/c nude (China)	6-8 wk N=6		Human non-small cell lung cancer (A549/CIS) 1x10^7 sc	D0 100mm^3	6x 2mg/kg iv every 3 days (D0,3,6,9,12,15) (cumulative 12 mg/kg) cisplatin prodrug multi-layered nano-platform	BW ↓30%, TV Blood: ↑ALT, ↑LDH, ↑BUN Tissue distribution: heart, kidney >liver, lung>spleen	D18<br><br>TV 1600mm3 (control)!	[14]
C57BL/6 (healthy)<br><br>athymic nude (China)	male 8wk N=6 female 7-8wk N=10		Human ovarian cancer cells (A2780) 1 x10^7 sc flank	D0 270mm^3 randomly divided	Healthy B6: 25mg/kg ip + daily ip (3x) vechicle (50% DMSO, saline)<br><br>tumor: 10mg/kg	Healthy: kidney: TUNEL, caspase3, Bax, Bcl (Western), mitochondrial ROS (IF) – Mito SOX ↑6x Cr, kidney (HE) score Tumor: TV, mortality 80% vs 10%	Healthy: 72h<br><br>Tumor: D7 Mortality 80% 8/10	[15]
BALB/c nude (China)	Female N=5	-d12	tumor cells MDA-MB-231 5x10^6 sc	80-150 mm^3 D0 randomly divided	5 x 1.5mg/kg every 3 days iv (cumulative 7.5 mg/kg)<br><br>Vs cisplatin complexes with naproxen	BW ≈ normal in all groups TS, tumor: COX-2, PD-L1(IHC)	D15 HI-nr Nr TV - 1200mm^3 (control)	[16]
BALB/c-nu nude (6wk)<br><br>C57BL/6 (8wk and 5 days)<br><br>(China)	Female N=5<br><br>mating N=20<br><br>Pups 5days old	D0	human ovarian cancer cells (C13) 2x10^6 sc flank<br><br>healthy mating newborn<br><br>ovary toxicity	D0<br><br>tumor 20mm^3 (D9)	Healthy/ tumor protocol: 3 x 5mg/kg/week for 3 wks (cumulative 15mg/kg) newborn: single 5 mg/kg ip<br><br>6 matings after protocol: 2 oestrus cycles measured prior; mating (N=20) N=3 ovary removed (D21) gene array;<br><br>vs cisplatin + melatonin (10 mg/kg daily)	Healthy: Ovary: weight ratio to body, morphology Expression of Msy2(Ybx2), P63 (Trp63) in ovary, TUNEL, gene array, MDA (IHC) Mating: pregnant rates, pup numbers (after 6 matings) Cisplatin administration destroyed regular oestrus cycles in mice and lipid metabolism, circadian behavior	D21 (D30) D3 (newborn) IHC the ovarian reserve<br><br>breeding, fertility, effect on cancer efficacy serum FSH assay – estrus cycle	[17]
BALB/c nude (China)	Female 6wks N=6	D0	lung tumor cells (MDA-MB-231) 1x10^6 sc<br><br>lung metastasis tumor cells (MDA-MB-231) 1x10^6 iv	D0	6 x 5mg/kg ip (twice weekly during 24 days) (cumulative 30 mg/kg) PP p/o daily for 24days Vs cisplatin + pivalopril (PP) improves anti-cancer efficacy of cisplatin – angiotensin-converting enzyme (ACE) inhibitor	D24 Blood: ↑Cr, ↑AST HE: lung, ↓kidney, liver, spleen, heart, Δ in all organs tumor TS: Ki67, VEGF (IHC) TUNEL Metastases - 8wks: lungs, pulmonary metastatic nodules were counted	D24 (sc)<br><br>8wks (iv) (metastasis)	[18]
BALB/c nude (China)	Male 4-6wks		A549 cells 5x10^6 sc	100-160mm^3 D0	2mg/kg ip per two days (cumulative ?4 or 14 mg/kg) 5% DMSO as control vs copper complexes	BW ↓8.4%, TS BW used as a parameter for systemic toxicity	D15	[19]
Athymic nu/nu (Mexico)	Female 6-7wks N=5		xenograft HeLa cells 5x10^6 sc back	150mm^3 D0 randomly divided	3x 3mg/kg ip weekly (cumulative 9mg/kg) Liposome nanoparticles to encapsulate cisplatin+milepristone and delivery to the target	BW ≈(no sign change among groups – variations observed), TV BW used as a measure for systemic toxicity	D50 (8wks)	[20]
athymic nude	female 4-5 wks	D0	orthotopic HPV negative xenograft (UM-SCC1-	D7	3x 4mg/kg over 15 days (cumulative 12mg/kg)	≈BW, TV ; other signs of treatment-related toxicities were recorded ?	D70; TV – BLS HEP? (fig 5)	[21]

(USA)	20g<br>N=5		luc) 1x10⁶ into the tongue		Vs novel targeted agent to inhibit DNA repair pathways – PARP1, DNA-PK inhibitors	BW used as a measure for systemic toxicity	D28 - TV max 1000mm³	
swiss albino (India)	Female<br>N=6	D0	Ehrlich ascites carcinoma (EAC) cells 1x10⁶ ip	D1	10x 5-50μM daily (from D1-D10)<br>20 μM (blood parameters)<br><br>Vs cytotoxic potential of xanthones present in plant *Swertia chirata*	4h fasted before euth<br>Blood:↓Hb, ↓RBC, ↑WBC, ↓platelet, ↑ALP, ↑AST, ↑ALT, ↑BUN, ↑Cr<br>survival test (N=10) D20-85	D15<br><br>EAC also affected all blood parameters – similar as cisplatin	[22]
BALB/c nude (China)	Male<br>5wks<br>N=6		Human gastric cancer cells (HGC-27) sc dorsal area	100-150 mm³<br>D0<br>randomly divided	5mg/kg every 2 days ip<br>(cumulative 10 mg/kg)<br>Vs hesperetin combined with cisplatin increase anticancer activity	BW↓(19%), TV<br>Blood: ≈BUN, Cr, ALT, AST<br>Tumors: TUNEL	D33<br><br>(fig6) max TV 1300mm³, 700mg	[23]
athymic CD-1 (Crl) (Italy)	Female<br>5wks<br>N=7		heterotopic Cal27 cells 6x10⁶ sc flank (N=7)<br>orthotopic GFP+/luc+ transfected Cal27 6x10⁴ into anterior tongue (N=10)	D8<br>became palpable randomly divided<br>D4	6x 1mg/kg ip (3x/week per 2 weeks)<br>(cumulative 6mg/kg) + CX<br><br>Vs valproic acid , cisplatin and cetuximab (three drug combinatorial strategy) increase antitumor activity	TV, tumor growth delay , BW↑<br>Tumor: IHC: Ki67, EGFR, AcH3<br><br>D21(N=3) blood: GOT, GPT, Cr<br>BSC imaging – survival curve (HEP)	D35<br>D21; N=3<br>HEP: BW↓10%<br>BSC >3000 (photons total count)	[24]
athymic BALB/cA nN-nu (China)	Female<br>4-5wks<br>N=5	D0	Ovarian cancer cells (ES2) 2x10⁶ ip - nodules in abdominal cavity	≈D7<br>palpable tumors	5x 2mg/kg every other day<br>(cumulative 10mg/kg)<br><br>Vs cisplatin + MAP30 protein has synergistic antitumor effects	BW≈, ascites tumors – BW?!<br>Nodules in abdomen: number, weight – 0,15g/tumor; 40-100 nodules in abdomen = 15g?+ fluid; average ascites volume 500-3000μL	?<br>(fig4; D35)	[25]
inbred albino Swiis (India)	male, female<br>10-12 wks<br>N=10	D0	Ascites Dalton's lymphoma tumor<br><br>Liver, kidney, testis toxicity	D10	8mg/kg ip single dose<br>cisplatin group did not receive saline insted rutin (6h prior cisplatin)?<br>vs rutin+cisplatin – against liver, nephro and testis toxicity	RW of organs: ↑kidney,↑ liver, ↑testis; Histology: kidney, liver, testis;<br>Blood (N=3): ALT↑(140%), AST↑(80%), ALP ↑(20%), BUN↑(20%), Cr↑(130%)	D15<br><br>in vitro hemolysis assay<br>sperm abnormality analysis	[26]
athymic nude mice (Turkey)	male<br>8wks<br>25-30g<br>N=18		neuroblastoma (C1300Myc positive) 1x10⁶ sc<br>cardiotoxicity	D10	16mg/kg single ip;<br>10days before po 0.75 saline hydration);<br>(dose - previous work from lab)<br>Vs acetyl-L-carnitine +cisplatin	heart : histology (no changes observed) IHC: ↑SOD-2,<br>≈ iNOS, COX-2, Bcl-2, caspase3<br>Blood: cTnI-Ultra↑	D18 euth (diethyl ether)	[27]
BALB/c nude (Korea)	male<br>6wk<br>N=5		Human bladder cancer EJ cells (MGH-U1) 1x10⁷ sc flank	250mm³	5mg/kg (single or multiple- nr!)<br><br>Vs Hizikia fusiforme celluclast extract antitumor efficacy	Cis: BW↓20%, TV (1g)<br>Tumor: HE and Ki67<br>Not measured in cisplatin group<br>Blood: ≈ALT, AST, ALP, BUN, Cr – not measured for cisplatin?<br>Histology: tumor, heart, kidney, liver, lungs	D20<br><br>↓Reporting quality!	[28]
BALB/c (Egypt)	Female<br>20-25g<br>N=10	D0	Ehrlich ascites tumor cells sc	30-50 mm³<br>D0	1mg/kg iv single – it refers to ref34 but dose is in ref 34 is different (4.5 mg/kg ip)<br><br>Vs gold nanorods cytotoxicity and genotoxicity	Tumor growth<br>Histology: liver, kidney: injuries<br>liver ↑SOD, ↓MDA<br>kidney ↓SOD, ≈MDA<br>tumor ↓MDA, ↓SOD,	D12<br><br>comet assay-genotoxicity	[29]
C57BL/6N<br><br>CD-1(ICR) Crl (Korea)	Female<br>8wks<br>N=18-22	D1	B16F10 melanoma cells 1.2x10⁵ sc femoral region<br>CD-1 -toxicity	D7	6x 3mg/kg ip (D7,8, 13,14,19,20 = 2x/ week for 3 wks)<br>(cumulative 18mg/kg), tumor bearing B6<br>healthy CD-1: toxicity -the same protocol of treatment<br>Vs herbal mixture -MH-30+cisplatin synergistic	Tumor: ↓weight (47.16%), spleen lymphocytes (NK cells, Tc) (D19)<br>Toxicity: weight- kidney ↓18%, liver ↓19%, BW ↓(10%)<br>Blood: ↑ALT; ↑AST, ↑BUN, ↑Cr;<br>Histology: no damage of liver, kidney	D23	[30]

					antitumor activity	– tubular necrosis		
BALB/c nude (China)	male 5wks N=5-10	D0	143B cells 5x10^6 sc flank SJSa-1 cells 5x10^5 into bone marrow cavity - femur		3mg/kg per wk for 3 weeks iv (cumulative 9 mg/kg) Vs fructose-coated angstrom silver antitumor activity and mechanism of action	BW, TV, lung, heart, kidney, liver, brain, spleen, tumor -TUNEL, Blood: ≈platelet, Hb, RBC, BUN, Cr, kidney weight ↓food and water consumption	D21 exp 1 ; D60 exp2 - survival HEP: when mice "deemed to unwell", or tumor exceed 2000mm^3	[31]
BALB/c nude (China)	Male 19-22g N=5	D0	tumor cells H157 1x 10^7 sc under arm	200-250mm^3 (D14) D0 Divided	0.7mM daily for 5 days ip (cumulative 3.5 mM) Vs frog skin-derived peptide dermaseptin-PP antitumor mechanisms – intratumoral injections!!?	BW ↓30%, dull skin Tumor: TV, weight, inhibitory rate Histology: tumor, metastatses: lung, liver ; spleen index ↓	D10	[32]
BALB/c nude (China)	Female 4wks 16±2g N=6		xenograft human breast carcinoma cells (MCF-7) 1x 10^6 sc oxter	50-100mm^3 D0	6x 2mg/kg iv every 4 days (cumulative 12 mg/kg)<br><br>Vs anticancer melatplatin prodrugs – combination of endocrine-, immune- and chemo-therapy	BW↓, TV (39-47% inhibition), Surival (33%), Histology: HE kidney, tumor, spleen; RW spleen↓(mg/g), ≈heart, kidney, liver, heart, lung; Cisplatin distribution: spleen (↑70µg/g), tumor (↓10µg/g), kidney (35 µg/g), liver (25 µg/g), heart (20 µg/g), lung (↓20 µg/g)	D23<br><br>Mortality: Dead mice HEP? BW↓ 20%	[33]
Kunming (China)	male 20-22g N=10	-d2	H22 20x 10^6 ip highly malignant peritoneal carcinosis	(48h) D0	9mg/kg single ip Vs N-acetyl-L-cysteine and selenium nanoparticles	BW (slow increase D0- D7) – peritoneal carcinosis mortality 10%D11,100% D16	D16	[34]
ICR (healthy)<br><br>Nude (China)	male, 7 wk 22-25g N=6-8<br><br>6wks		ICR : healthy - nephrotoxicity Nude: Non-small cell lung cancer (H460) or Human colon cancer (HCT116) 1x10^7 sc/site rear flank	200-300mm^3	10 mg/kg single iv<br><br>Vs L-tetrahydropalmatine against cisplatin nephrotoxicity	BW↓20%; Kidney: histology- injury, mRNA Kim-1 ↑60x, Lcn-2 ↑98x cisplatin concentration: kidney (6µg/g), urin (250µg/72h), tumor (>1µg/g), kidney (4 µg/g) blood: BUN↑3x (healthy), ↑1.5-1.9x (tumor), ≈Cr, uric acid,	72 h<br><br>healthy and tumor bearing mice Mouse behavior, BW, TV monitored every day	[35]
athymic nude (Crl) (USA)	female 4wk N=8-10	D0	HPV-negative UM-SCC1-luc 1x10^5 oral tongue (BLS imaging) (orthotopic) HPV positive UM-SCC47 3x10^6 sc flank (heterotopic)	D5	4mg/kg per week for 3 wks (cumulative 12 mg/kg) Head and neck squamous cell carcinomas<br><br>Vs CHK1/2 inhibitor prexasertib +cis +radiotheraphy improves antitumor activity	TV, tumor growth, BW	up to D100 HEP 1600x BSL up to D65 1000mm^3	[36]
Nude (Turkey)	Female 6-8 wks 24g N=6	D0	SKBR3 and MDA-MB-453 1x 107 sc flank	50-100mm3 D32	6 mg/kg single ip Vs cisplatin-combination therapy pretreatment with nanoparticles SP-AH/MIR376B increased antitumor activity	TV	D36	[37]
BALB/c (China)	N=14		H460 and LTEP-A-2 implantation of tumor pieces into anterior axilla	100-200mm^3 D0 randomly divided	7x 6 mg/kg (every three days) iv (cumulative 42 mg/kg) ?<br><br>Vs diplatin – a novel water-soluble Pt complex	TV – growth, TUNEL<br><br>Water solubility of cisplatin =2.50mg/ml,	D22	[38]

Legend: MTD-maximum tolerated dose; HEP- humane endpoint (mice were humanely euthanized); BW-body weight; TS-tumor size; TV-tumor volume; RW –relative weight of organ; BLS – bioluminescence imaging; N-number; M-mortality; ip-intraperiteneally; iv- intravenously; sc-subcutaneously; D/d-day; Δ-denotes alterations; ≈-ns; ↓-decrease; ↑-increase; BUN-blood urea nitrogen; Cr-serum creatinine; GFR-glomerular filtration rate; IHC-immunohistochemistry; HE-haematoxylin eosin; IF-immunofluorescence; WBC-white blood cells; RBC-red blood cells; Hb-hemoglobine; ALT-alanine transaminase; AST-aspartate transaminase; ALP-alkaline phosphatase; LDH-lactate dehydrogenase; TBILS-total bilirubin; CytC-cytochrome c; COX-2-cyclooxigenase2; ROS-reactive oxygen species; MDA-malondialdehyde; SOD-superoxide dismutase;

References

1. Naik, A.V.; Dessai, S.N.; Sellappan, K. Antitumour activity of Annona muricata L. leaf methanol extracts against Ehrlich Ascites Carcinoma and Dalton's Lymphoma Ascites mediated tumours in Swiss albino mice. *Libyan J Med* **2021**, *16*, 1846862, doi:10.1080/19932820.2020.1846862.
2. Zhang, M.; Hagan, C.T.; Foley, H.; Tian, X.; Yang, F.; Au, K.M.; Mi, Y.; Medik, Y.; Roche, K.; Wagner, K.; et al. Co-delivery of etoposide and cisplatin in dual-drug loaded nanoparticles synergistically improves chemoradiotherapy in non-small cell lung cancer models. *Acta Biomater* **2021**, *124*, 327-335, doi:10.1016/j.actbio.2021.02.001.
3. Blättner, S.; Das, S.; Paprotka, K.; Eilers, U.; Krischke, M.; Kretschmer, D.; Remmele, C.W.; Dittrich, M.; Müller, T.; Schuelein-Voelk, C.; et al. Staphylococcus aureus Exploits a Non-ribosomal Cyclic Dipeptide to Modulate Survival within Epithelial Cells and Phagocytes. *PLoS Pathog* **2016**, *12*, e1005857, doi:10.1371/journal.ppat.1005857.
4. Zhang, H.; Almuqbil, R.M.; Alhudaithi, S.S.; Sunbul, F.S.; da Rocha, S.R.P. Pulmonary administration of a CSF-1R inhibitor alters the balance of tumor-associated macrophages and supports first-line chemotherapy in a lung cancer model. *Int J Pharm* **2021**, *598*, 120350, doi:10.1016/j.ijpharm.2021.120350.
5. Wu, W.J.; Tang, Y.F.; Dong, S.; Zhang, J. Ginsenoside Rb3 Alleviates the Toxic Effect of Cisplatin on the Kidney during Its Treatment to Oral Cancer via TGF-. *Evid Based Complement Alternat Med* **2021**, *2021*, 6640714, doi:10.1155/2021/6640714.
6. Wang, B.; Hu, W.; Yan, H.; Chen, G.; Zhang, Y.; Mao, J.; Wang, L. Lung cancer chemotherapy using nanoparticles: Enhanced target ability of redox-responsive and pH-sensitive cisplatin prodrug and paclitaxel. *Biomed Pharmacother* **2021**, *136*, 111249, doi:10.1016/j.biopha.2021.111249.
7. Arita, M.; Watanabe, S.; Aoki, N.; Kuwahara, S.; Suzuki, R.; Goto, S.; Abe, Y.; Takahashi, M.; Sato, M.; Hokari, S.; et al. Combination therapy of cisplatin with cilastatin enables an increased dose of cisplatin, enhancing its antitumor effect by suppression of nephrotoxicity. *Sci Rep* **2021**, *11*, 750, doi:10.1038/s41598-020-80853-6.
8. Zhang, Q.; Lu, Q.B. New combination chemotherapy of cisplatin with an electron-donating compound for treatment of multiple cancers. *Sci Rep* **2021**, *11*, 788, doi:10.1038/s41598-020-80876-z.
9. Hadi, T.; Ramseyer, C.; Gautier, T.; Bellaye, P.S.; Lopez, T.; Schmitt, A.; Foley, S.; Yesylevskyy, S.; Minervini, T.; Douhard, R.; et al. Lipoproteins LDL versus HDL as nanocarriers to target either cancer cells or macrophages. *JCI Insight* **2020**, *5*, doi:10.1172/jci.insight.140280.
10. Hazem, R.M.; Mohamed, A.A.; Ghareb, N.; Mehanna, E.T.; Mesbah, N.M.; Abo-Elmatty, D.M.; Elgawish, M.S. Anti-cancer activity of two novel heterocyclic compounds through modulation of VEGFR and miR-122 in mice bearing Ehrlich ascites carcinoma. *Eur J Pharmacol* **2021**, *892*, 173747, doi:10.1016/j.ejphar.2020.173747.
11. Zhang, Y.; Dong, Y.; Fu, H.; Huang, H.; Wu, Z.; Zhao, M.; Yang, X.; Guo, Q.; Duan, Y.; Sun, Y. Multifunctional tumor-targeted PLGA nanoparticles delivering Pt(IV)/siBIRC5 for US/MRI imaging and overcoming ovarian cancer resistance. *Biomaterials* **2021**, *269*, 120478, doi:10.1016/j.biomaterials.2020.120478.
12. Zhang, M.; Liu, Q.; Cao, C.; Liu, X.; Li, G.; Xu, C.; Zhang, X. Enhanced antitumor effects of follicle-stimulating hormone receptor-mediated hexokinase-2 depletion on ovarian cancer mediated by a shift in glucose metabolism. *J Nanobiotechnology* **2020**, *18*, 161, doi:10.1186/s12951-020-00720-4.
13. Cao, Z.; Li, W.; Liu, R.; Li, C.; Song, Y.; Liu, G.; Chen, Y.; Lu, C.; Lu, A.; Liu, Y. pH-Responsive Fluorescence Enhanced Nanogel for Targeted Delivery of AUR and CDDP Against Breast Cancer. *Int J Nanomedicine* **2020**, *15*, 8369-8382, doi:10.2147/IJN.S274842.
14. Liang, C.J.; Shen, W.C.; Chang, F.B.; Wu, V.C.; Wang, S.H.; Young, G.H.; Tsai, J.S.; Tseng, Y.C.; Peng, Y.S.; Chen, Y.L. Endothelial Progenitor Cells Derived From Wharton's Jelly of Human Umbilical Cord Attenuate Ischemic Acute Kidney Injury by Increasing Vascularization and Decreasing Apoptosis, Inflammation, and Fibrosis. *Cell Transplant* **2015**, *24*, 1363-1377, doi:10.3727/096368914X681720.
15. Sun, Z.; Xu, S.; Cai, Q.; Zhou, W.; Jiao, X.; Bao, M.; Yu, X. Wnt/β-catenin agonist BIO alleviates cisplatin-induced nephrotoxicity without compromising its efficacy of anti-proliferation in ovarian cancer. *Life Sci* **2020**, *263*, 118672, doi:10.1016/j.lfs.2020.118672.
16. Jin, S.; Muhammad, N.; Sun, Y.; Tan, Y.; Yuan, H.; Song, D.; Guo, Z.; Wang, X. Multispecific Platinum(IV) Complex Deters Breast Cancer via Interposing Inflammation and Immunosuppression as an Inhibitor of COX-2 and PD-L1. *Angew Chem Int Ed Engl* **2020**, *59*, 23313-23321, doi:10.1002/anie.202011273.
17. Huang, J.; Shan, W.; Li, N.; Zhou, B.; Guo, E.; Xia, M.; Lu, H.; Wu, Y.; Chen, J.; Wang, B.; et al. Melatonin provides protection against cisplatin-induced ovarian damage and loss of fertility in mice. *Reprod Biomed Online* **2021**, *42*, 505-519, doi:10.1016/j.rbmo.2020.10.001.
18. Fei, L.; Huimei, H.; Dongmin, C. Pivalopril improves anti-cancer efficiency of cDDP in breast cancer through inhibiting proliferation, angiogenesis and metastasis. *Biochem Biophys Res Commun* **2020**, *533*, 853-860, doi:10.1016/j.bbrc.2020.07.059.
19. Gul, N.S.; Khan, T.M.; Chen, M.; Huang, K.B.; Hou, C.; Choudhary, M.I.; Liang, H.; Chen, Z.F. New copper complexes inducing bimodal death through apoptosis and autophagy in A549 cancer cells. *J Inorg Biochem* **2020**, *213*, 111260, doi:10.1016/j.jinorgbio.2020.111260.

20. Ledezma-Gallegos, F.; Jurado, R.; Mir, R.; Medina, L.A.; Mondragon-Fuentes, L.; Garcia-Lopez, P. Liposomes Co-Encapsulating Cisplatin/Mifepristone Improve the Effect on Cervical Cancer: In Vitro and In Vivo Assessment. *Pharmaceutics* **2020**, *12*, doi:10.3390/pharmaceutics12090897.
21. Zeng, L.; Boggs, D.H.; Xing, C.; Zhang, Z.; Anderson, J.C.; Wajapeyee, N.; Veale, C.; Bredel, M.; Shi, L.Z.; Bonner, J.A.; et al. Combining PARP and DNA-PK Inhibitors With Irradiation Inhibits HPV-Negative Head and Neck Cancer Squamous Carcinoma Growth. *Front Genet* **2020**, *11*, 1036, doi:10.3389/fgene.2020.01036.
22. Barua, A.; Choudhury, P.; Mandal, S.; Panda, C.K.; Saha, P. Therapeutic potential of xanthones from. *Indian J Med Res* **2020**, *152*, 285-295, doi:10.4103/ijmr.IJMR_1153_18.
23. He, P.; Ma, J.; Liu, Y.; Deng, H.; Dong, W. Hesperetin Promotes Cisplatin-Induced Apoptosis of Gastric Cancer. *Front Pharmacol* **2020**, *11*, 1326, doi:10.3389/fphar.2020.01326.
24. Iannelli, F.; Zotti, A.I.; Roca, M.S.; Grumetti, L.; Lombardi, R.; Moccia, T.; Vitagliano, C.; Milone, M.R.; Ciardiello, C.; Bruzzese, F.; et al. Valproic Acid Synergizes With Cisplatin and Cetuximab. *Front Cell Dev Biol* **2020**, *8*, 732, doi:10.3389/fcell.2020.00732.
25. Chan, D.W.; Yung, M.M.; Chan, Y.S.; Xuan, Y.; Yang, H.; Xu, D.; Zhan, J.B.; Chan, K.K.; Ng, T.B.; Ngan, H.Y. MAP30 protein from Momordica charantia is therapeutic and has synergic activity with cisplatin against ovarian cancer in vivo by altering metabolism and inducing ferroptosis. *Pharmacol Res* **2020**, *161*, 105157, doi:10.1016/j.phrs.2020.105157.
26. Prasad, R.; Prasad, S.B. Histoprotective effect of rutin against cisplatin-induced toxicities in tumor-bearing mice: Rutin lessens cisplatin-induced toxicities. *Hum Exp Toxicol* **2021**, *40*, 245-258, doi:10.1177/0960327120947793.
27. Bayrak, S.; Aktaş, S.; Altun, Z.; Çakir, Y.; Tütüncü, M.; Kum Özşengezer, S.; Yilmaz, O.; Olgun, N. Antioxidant effect of acetyl-l-carnitine against cisplatin-induced cardiotoxicity. *J Int Med Res* **2020**, *48*, 300060520951393, doi:10.1177/0300060520951393.
28. Song, J.H.; Won, S.Y.; Hwang, B.; Jung, S.; Choi, C.; Park, S.S.; Choi, Y.H.; Kim, W.J.; Moon, S.K. In Vitro and In Vivo Antitumor Efficacy of. *Nutrients* **2020**, *12*, doi:10.3390/nu12072159.
29. Monem, A.S.; Sayed, F.A.; Rageh, M.M.; Mohamed, N. Cytotoxicity and genotoxicity of gold nanorods assisted photothermal therapy against Ehrlich carcinoma in-vivo. *Life Sci* **2020**, *257*, 118108, doi:10.1016/j.lfs.2020.118108.
30. Park, H.R.; Jo, S.K.; Cho, H.H.; Jung, U. Synergistic Anti-cancer Activity of MH-30 in a Murine Melanoma Model Treated With Cisplatin and its Alleviated Effects Against Cisplatin-induced Toxicity in Mice. *In Vivo* **2020**, *34*, 1845-1856, doi:10.21873/invivo.11979.
31. Hu, X.K.; Rao, S.S.; Tan, Y.J.; Yin, H.; Luo, M.J.; Wang, Z.X.; Zhou, J.H.; Hong, C.G.; Luo, Z.W.; Du, W.; et al. Fructose-coated Angstrom silver inhibits osteosarcoma growth and metastasis via promoting ROS-dependent apoptosis through the alteration of glucose metabolism by inhibiting PDK. *Theranostics* **2020**, *10*, 7710-7729, doi:10.7150/thno.45858.
32. Dong, Z.; Hu, H.; Yu, X.; Tan, L.; Ma, C.; Xi, X.; Li, L.; Wang, L.; Zhou, M.; Chen, T.; et al. Novel Frog Skin-Derived Peptide Dermaseptin-PP for Lung Cancer Treatment:. *Front Chem* **2020**, *8*, 476, doi:10.3389/fchem.2020.00476.
33. Song, X.Q.; Liu, R.P.; Wang, S.Q.; Li, Z.; Ma, Z.Y.; Zhang, R.; Xie, C.Z.; Qiao, X.; Xu, J.Y. Anticancer Melatplatin Prodrugs: High Effect and Low Toxicity, MT1-ER-Target and Immune Response. *J Med Chem* **2020**, *63*, 6096-6106, doi:10.1021/acs.jmedchem.0c00343.
34. Zhao, G.; Dong, R.; Teng, J.; Yang, L.; Liu, T.; Wu, X.; He, Y.; Wang, Z.; Pu, H.; Wang, Y. -Acetyl-l-cysteine Enhances the Effect of Selenium Nanoparticles on Cancer Cytotoxicity by Increasing the Production of Selenium-Induced Reactive Oxygen Species. *ACS Omega* **2020**, *5*, 11710-11720, doi:10.1021/acsomega.0c01034.
35. Li, C.; Li, L.; Yi, Y.; Wang, W.; Yuan, J.; Tan, F.; Fang, D.; Zeng, S.; Zhou, H.; Jiang, H. L-tetrahydropalmatine attenuates cisplatin-induced nephrotoxicity via selective inhibition of organic cation transporter 2 without impairing its antitumor efficacy. *Biochem Pharmacol* **2020**, *177*, 114021, doi:10.1016/j.bcp.2020.114021.
36. Zeng, L.; Nikolaev, A.; Xing, C.; Della Manna, D.L.; Yang, E.S. CHK1/2 Inhibitor Prexasertib Suppresses NOTCH Signaling and Enhances Cytotoxicity of Cisplatin and Radiation in Head and Neck Squamous Cell Carcinoma. *Mol Cancer Ther* **2020**, *19*, 1279-1288, doi:10.1158/1535-7163.MCT-19-0946.
37. Unal, O.; Akkoc, Y.; Kocak, M.; Nalbat, E.; Dogan-Ekici, A.I.; Yagci Acar, H.; Gozuacik, D. Treatment of breast cancer with autophagy inhibitory microRNAs carried by AGO2-conjugated nanoparticles. *J Nanobiotechnology* **2020**, *18*, 65, doi:10.1186/s12951-020-00615-4.
38. Wang, L.; Zeng, X.; Ye, J.; Chen, X.; Lin, X. Diplatin, a novel water-soluble platinum complex, inhibits lung cancer growth via augmentation of Fas-mediated apoptosis. *Eur J Pharmacol* **2020**, *879*, 173128, doi:10.1016/j.ejphar.2020.173128.
